# Inhaled magnesium versus inhaled salbutamol in rescue treatment for moderate and severe asthma exacerbations in pediatric patients

**DOI:** 10.1016/j.jped.2024.03.012

**Published:** 2024-04-30

**Authors:** Michelle Siqueira Debiazzi, Rossano César Bonatto, Fábio Joly Campos, Joelma Gonçalves Martin, José Roberto Fioretto, Maria Letícia das Neves Hansen, Arthur Martins de Araújo Luz, Haroldo Teófilo de Carvalho

**Affiliations:** Faculdade de Medicina de Botucatu, Departamento de Pediatria, Universidade Estadual Paulista “Júlio de Mesquita Filho” (UNESP), Botucatu, SP, Brazil

**Keywords:** Exacerbation, Asthma, Pediatrics

## Abstract

**Objective:**

To compare the effectiveness of inhaled Magnesium Sulfate associated with Salbutamol versus Inhaled Salbutamol alone in patients with moderate and severe asthma exacerbations.

**Method:**

Clinical, prospective and randomized study with patients between 3 and 14 years of age divided into two groups: one to receive inhaled salbutamol associated with magnesium sulfate (GSM), the other to receive inhaled salbutamol alone (GS). The sample consisted of 40 patients, 20 patients in each group. Severity was classified using the modified Wood-Downes score, with values between 4 and 7 classified as moderate and 8 or more classified as severe.

**Results:**

Post-inhalation scores decreased both in patients who received salbutamol and magnesium and in those who received salbutamol alone, with no statistically significant difference between the groups.

**Conclusions:**

Despite the benefits when administered intravenously, inhalation of the drug alone or in combination did not reduce the severity of the exacerbation.

## Introduction

Asthma treatment is classically guideline-based and focuses on symptom intensity and severity, and choosing the most appropriate therapy to control and reduce the risk of exacerbations. However, regardless of severity and despite adequate treatment, many children experience inflammatory process exacerbation, symptom intensification, and loss of disease control requiring hospitalization, sometimes in intensive care, the use of intravenous medications and mechanical ventilation, with a progressive loss in long-term lung function.^1^^,^^2^

The most common triggers are respiratory infections, especially those caused by viruses. In schoolchildren, the rate of hospital admission for asthma exacerbations correlates with seasonality and the consequent increase in acute respiratory infections between the months of September and December, and again in spring. Similar spikes in hospitalization for asthma exacerbations in adults are also seen at these times of year.^3^, ^4^, ^5^

Factors such as allergic sensitization, changes in immunity, antiviral response, bacterial infections, and exposure to allergens increase the susceptibility or risk of exacerbations and are directly related to the success of asthma maintenance treatment.^6^, ^7^, ^8^, ^9^, ^10^, ^11^, ^12^

Early recognition and intervention are fundamental for controlling exacerbations; however, a limited number of medications are supported in the literature for use at this stage. Bronchodilators (short-acting β-2 agonists and long-acting β-2 agonists), anticholinergics (ipratropium bromide), inhaled or systemic corticosteroids and the combination of inhaled corticosteroids with long-acting β-2 are the main medications used to control exacerbations, with adjuvant therapies such as magnesium sulfate, intravenous bronchodilators and methylxanthines reserved for refractory cases or where severe acute asthma is diagnosed.^13^, ^14^, ^15^, ^16^, ^17^, ^18^, ^19^, ^20^, ^21^

Whilst intravenous magnesium sulfate is frequently given to pediatric patients in emergency units, as its effectiveness has already been demonstrated in both practice and studies on this subject, its inhalation in association with bronchodilators has only been used in asthma exacerbations in adult patients. However, data available in the literature for children is insufficient, with most studies presenting different methods, interventions, or comparisons. However, there does appear to be an additive effect in this population when it is inhaled in combination with another drug.^22^, ^23^, ^24^

We, therefore, compared the effectiveness of nebulization using salbutamol with magnesium sulfate versus salbutamol alone in moderate and severe asthma exacerbations treated in the pediatric emergency department.

## Patients and methods

This is a prospective, randomized, clinical, pilot study approved by the ethics committee for human research (protocol: 5948,317), involving patients with asthma exacerbation, carried out over a 1-year period in the pediatric emergency department of a large hospital.

The modified Wood-Downes score was applied to classify severity (Table S1 - Supplementary Material).^25^ Patients who scored between 5 and 7 points were classified as moderate and those scoring 8 points or more were classified as severe; patients were subsequently randomized into two groups: Magnesium associated with salbutamol Group (GMS) and Salbutamol only Group (GS). GMS patients received 2.5 mg of nebulized salbutamol (ages 2–5 years) or 5 mg (ages ≥ 6 years) associated with 150 mg (1.5 mL) of 10 % magnesium sulfate (ages 2–6 years) and 250 mg (2.5 mL) of 10 % magnesium sulfate (ages 6–14 years), average doses found in pediatric literature which was scarce.^23^^,^^24^ GS patients received salbutamol only at the doses mentioned above.

Patients between 3 and 14 years old participated in the study. They were randomized using an electronically generated sequence from the https://www.randomizer.org website into the two study groups described above. Patients with mild asthma exacerbation, those with tracheostomy, upper and lower airway anomalies, laryngotracheomalacia, neuromuscular diseases, and rib cage malformations were excluded.^26^

## Results

After randomization and application of exclusion criteria, 40 patients diagnosed with moderate and severe asthma exacerbation were recruited. In GS, 5 (25 %) were between 3 and 6 years old; 7 (35 %) between 6 and 10 years old; and 8 (40 %) between 10 and 14 years old. Regarding race, 11 (55 %) declared themselves white, 5 (25 %) black, and 4 (20 %) of mixed race. Eleven (55 %) were male and 9 (45 %) were female; eight patients (40 %) were continuously using inhaled Beclomethasone corticosteroid (Available in the Brazilian Public Health Network). In GSM, 6 (30 %) were between 3 and 6 years old; 9 (45 %) between 6 and 10 years old; and 5 (25 %) between 10 and 14 years old. Regarding race, 12 (60 %) declared themselves white, 6 (30 %) black, and 2 (10 %) of mixed race. Eight (40 %) were male and 12 (60 %) were female; 5 patients (25 %) used Beclomethasone continuously.

The first step in statistical analysis was to evaluate whether the score decreased after inhalation in both groups and then whether the decrease was greater in GSM than GS. Before comparing samples, it was necessary to perform the Shapiro–Wilk normality test. The normality pre-test results are shown in Table 1 and distributions are shown in Figs. S1 and S2 (Supplementary Material).

**Table 1:** Results of the Shapiro–Wilk normality test with a 5 % significance.

Variable	W statistic	*p*-value
Score before	0.7780	< 0.001
Score after	0.8515	< 0.001

The Wilcoxon *t*-test was used to compare scores before and after inhalation. This test is used in situations where the authors want to compare paired samples, such as with patients who have a score measured before and a score measured after. The Mann–Whitney U test was used to compare the decrease in scores between groups, which is appropriate for situations where samples are independent, as is the case with groups containing different patients. Test results are shown in Table 2 and presented in Fig. S3 (Supplementary Material).

**Table 2:** Comparison of scores before and after reduction and of scores between groups with a 5 % significance.

Comparison	V statistic	*p*-value
GS: before and after	190	< 0.001
GSM: before and after	153	< 0.001

Comparison	W statistic	*p*-value

Score Reduction: GS × GSM	193.5	0.8684

The Wilcoxon test resulted in a *p*-value < 0.05, therefore, the authors can consider the score after inhalation to be significantly lower than the score before in both groups. However, when the authors compare the decrease in scores between the groups, we do not have a statistically significant difference. This effect may be influenced by age group, which in the experimental design are blocks that can promote variance. The authors therefore applied two-way analysis to verify this effect.

Two-way Analysis of Variance (ANOVA) was conducted to test the effect of patient age on interaction with treatment. To ensure test robustness, the normality of residuals was confirmed (*W* = 0.977 and *p* = 0.595). If ANOVA produced significant results, a multiple comparison post-test would be conducted. The result of the two-way ANOVA is shown in Table 3 and presented in Fig. S4 (Supplementary Material).

**Table 3:** Results of two-way ANOVA with 5 % significance.

	Degree of liberty	SQ	QM	F	*p*-value
Group	1	0.03	0.025	0.010	0.920
Age	2	10.74	5.369	2.223	0.124
Group × Age	2	1.09	0.543	0.225	0.800
Residuals	34	82.13	2.415		

SQ, Sum of squares; QM, Mean of squares.

Two-way ANOVA did not obtain any *p*-value < 0.05. Therefore, there is not enough evidence to state that there was an effect from treatment, age, or the interaction between treatment and age on score decrease. Considering that this is a pilot study, the authors can hypothesize that a larger sample size will be capable of demonstrating such effects.

The authors applied Fisher's exact test to assess whether there is an association between treatment received and possible outcomes (improvement, hospitalization, intubation, and medications). To evaluate the association between treatment and patient improvement, the authors created a binary variable called “Improvement”: ‘0’, when the Wood-Downes score after inhalation is equal to or greater than before, and ‘1’, when the Wood-Downes score after inhalation is lower than before. The other variables were already binary and were used as provided. Fisher's test results are shown in Table 2S (Supplementary Material).

Fisher's test did not return any *p*-value < 0.05, therefore, there is no evidence of the association between treatment received by patients in either group and possible evaluated outcomes.

The authors applied Point-Biserial Correlation to evaluate the correlation between the decrease in score, patient characteristics, and possible outcomes. The analysis demonstrated, with *p* < 0.05, that patients over 10 years old tended to have a smaller decrease in the score. Home use of beclomethasone correlated with a greater decrease in the score, just as a decrease in score was related to fewer ICU admissions. It is noteworthy that correlation analysis does not have the power to infer causality, however, it highlighted variables for investigation in future studies (Table 4 and Figs. S5, S6 and S7 - Supplementary Material).

**Table 4:** Point-Bisseral Correlation between score reduction, patient characteristics and possible outcomes with 5 % significance.

Variable	Correlation coefficient r	*p*-value
Groups	0.016	0.9204
3–6 years old	0.074	0.6501
6–10 years old	0.246	0.1254
> 10 years old	−0.328	0.0386
Gender	−0.123	0.4484
Medications	−0.189	0.2437
Beclomethasone use	0.334	0.0350
ICU admission	−0.440	0.0046
Orotracheal intubation	−0.258	0.1077

## Discussion

Magnesium sulfate was initially considered for the treatment of asthma around 60 years ago, and since the 2015 GINA, has been indicated as an additive, via inhalation, in cases of severe asthma in adults. Of treatment options available during acute exacerbations, the effectiveness of intravenous MgSO_4_ has already been demonstrated, however little is known about its effectiveness when inhaled by children and adolescents.^20^

In acute therapy, magnesium sulfate is often given intravenously to reduce fatal outcomes while waiting for corticosteroid administration to take effect. Magnesium sulfate is an effective muscle relaxant commonly used to reduce asthma severity. It can relax the smooth muscle by two mechanisms: inhibiting the interaction between calcium and myosin, and cholinergic neuromuscular transmission is responsible for muscle fiber excitability.^21^^,^^22^

Similar studies have demonstrated that inhaled magnesium sulfate does not appear to act directly as a bronchodilator in children with asthma; however, in moderate and severe exacerbations, its response appears to be similar to that of albuterol, although the magnitude and duration of its effect may not be so marked. The present results contradicted this hypothesis, as magnesium sulfate did not exert an additional effect when inhaled. However, it did not cause side effects worthy of being reported by patients or their guardians.^23^^,^^24^

A prospective double-blinded randomized control study with 33 children compared the efficacy of magnesium sulfate versus inhaled Fenoterol in combination with ipratropium bromide found no statistically significant difference between the two study groups, as well as in length of hospital stay. The medication was safe, with no patient experiencing side effects.^25^^,^^26^

When the authors compared the association between inhaled magnesium and bronchodilators, and intubations and ICU admissions, we also found results similar to ours in literature, with a reduction in these rates as severity decreases. However, a study comparing this combination to placebo did not show a reduction in length of hospital stay, a variable not measured in this sample.^27^^,^^28^

A review including six studies involving 296 patients showed that magnesium sulfate associated with inhaled bronchodilators in treating exacerbations appeared to bring benefits in terms of improving lung function in patients with severe acute asthma, and a tendency towards reducing hospital admissions. However, Mega et al. did not find this effect when evaluating lung function in moderate and severe cases for patients who received inhaled magnesium sulfate.^28^^,^^29^

Another combination studied was inhaled magnesium sulfate with albuterol versus placebo. As with combinations with salbutamol, this association did not significantly reduce the severity or hospitalization rate for asthma exacerbations.^30^

Despite the known benefits of magnesium sulfate when administered intravenously in children and adolescents, inhalation of this drug, alone or in combination, did not reduce the severity of moderate and severe asthma exacerbations, however, reduction in severity score seems to be related to fewer intubations and admissions to pediatric intensive care units.

Despite being a pilot study, the number of patients recruited, the number of studies available in the pediatric literature, as well as the short period of follow-up of these patients were the main limitations. Further studies with large randomized samples and longer evaluation periods are needed to support or refute the present results.

## Conflicts of interest

The authors declare no conflicts of interest, and they each contributed fully to the preparation of this manuscript.

## Appendix Supplementary materials

Image, application 1
