# Supplementary material for: Inhaled magnesium versus inhaled salbutamol in rescue treatment for moderate and severe asthma exacerbations in pediatric patients
Source: J Pediatr (Rio J). 2024 Apr 30;100(5):539–43. doi: 10.1016/j.jped.2024.03.012 (PMC11361882; doi:10.1016/j.jped.2024.03.012)

**JPED-D-24-00062 –Supplementary Material**

**Table S1** Wood-Downes Score.

| **Table S1 - Wood-Downes Score** | | | |
| --- | --- | --- | --- |
| Evaluated Criteria | Score: 1 - 3: mild, 4 - 7: moderate, ≥ 8: severe | | |
|  | **0** | **1** | **2** |
| Level of Consciousness | Oriented | Agitated | Sleepy |
| Cyanosis or Saturation | Acyanotic  or Saturation > 94% | Peripheral Cyanosis  or Saturation < 94% | Central Cyanosis  or Saturation < 90% |
| Vesicular Murmur | Normal | Reduced | Abolished |
| Expiratory Wheezing | Absent | Sparse | Diffuse |
| Respiratory Discomfort | Absent | Mild | Marked |
| Adapted from Reference 25. | | | |

**Table S2** Association between treatment received and patient improvement, hospitalisation, intubation, and medication use with 5% significance.

| Table S2 Association between treatment received and patient improvement, hospitalisation, intubation, and medication use with 5% significance. | | |
| --- | --- | --- |
| Variable | Odds ratio | p-value |
| Improvement | 3.259 | 0.605 |
| Hospitalisation | 0.282 | 0.105 |
| Intubation | 1.000 | 1.000 |
| Medications | 0.527 | 1.805 |

**Figure S1** Distribution before treatment (Supplementary Material).


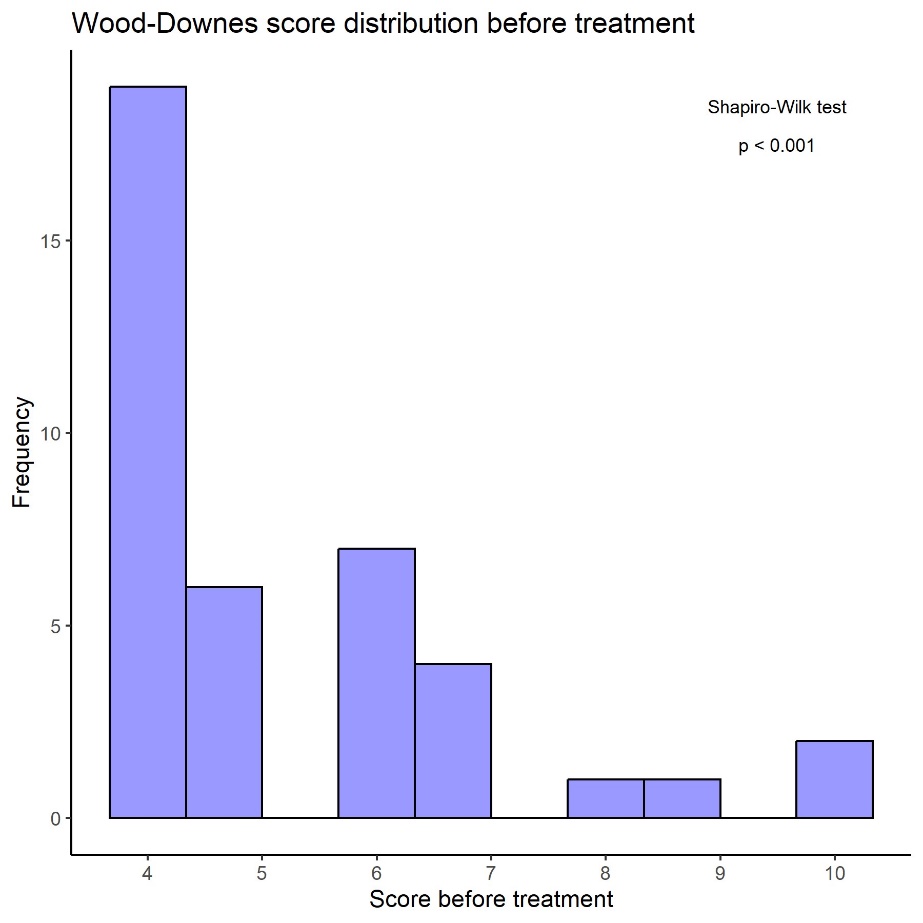


**Figure S2** Distribution after treatment.


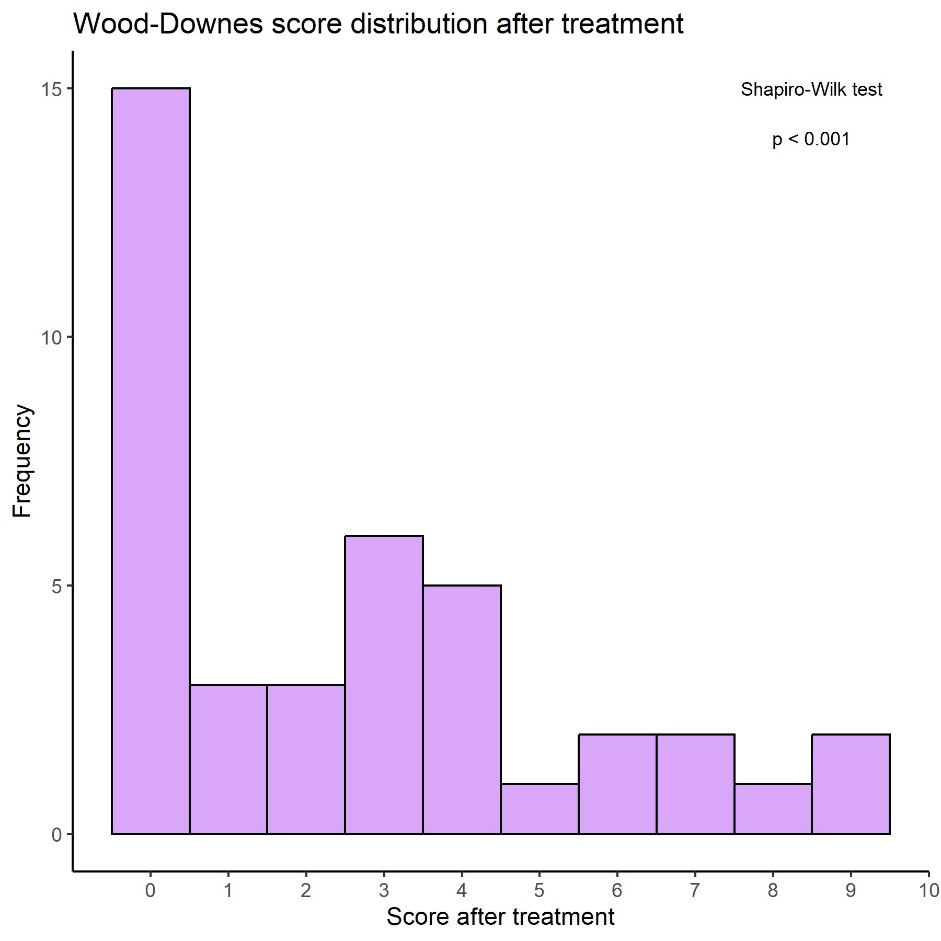


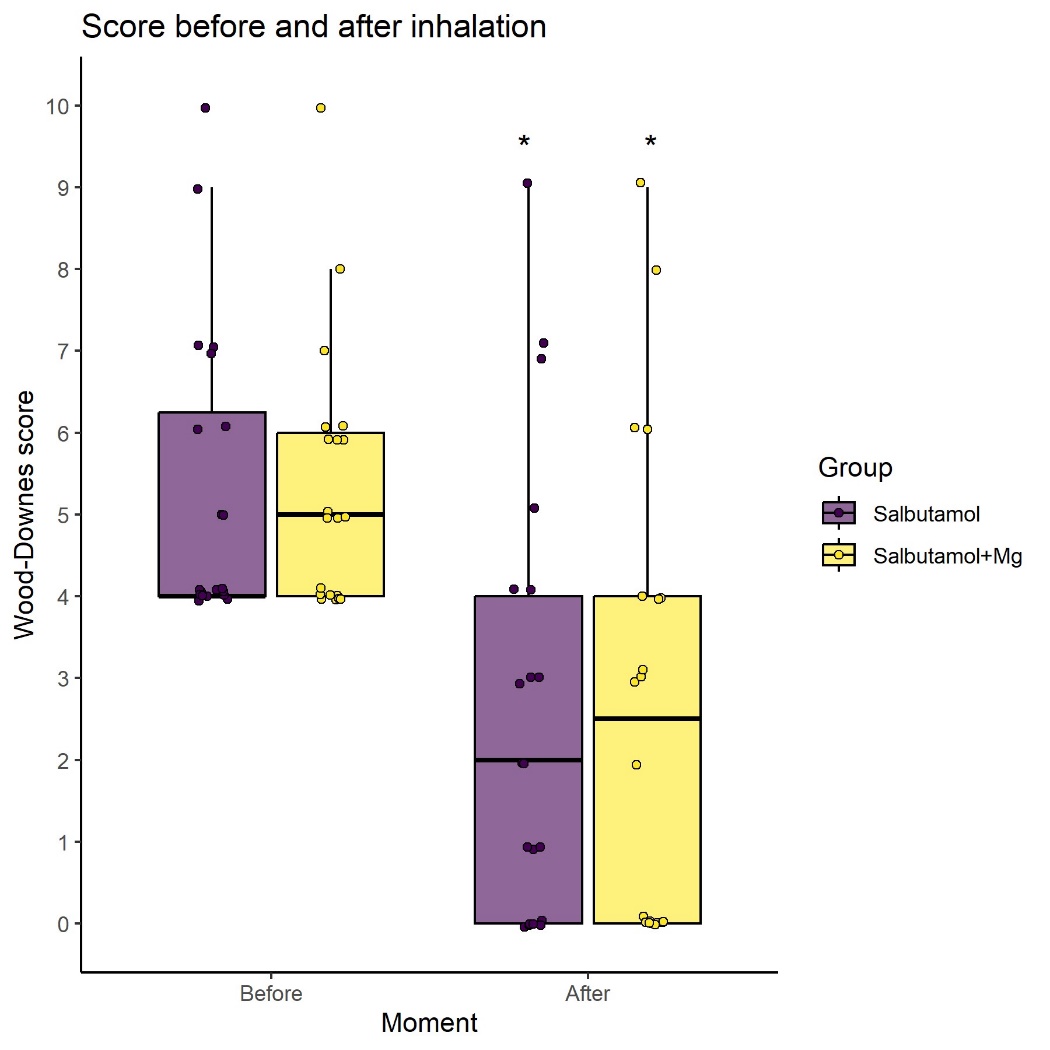


**Figure S3** Wilcoxon Test (Supplementary Material).

**Figure S4** Analysis of Variance (ANOVA) - Supplementary Material.


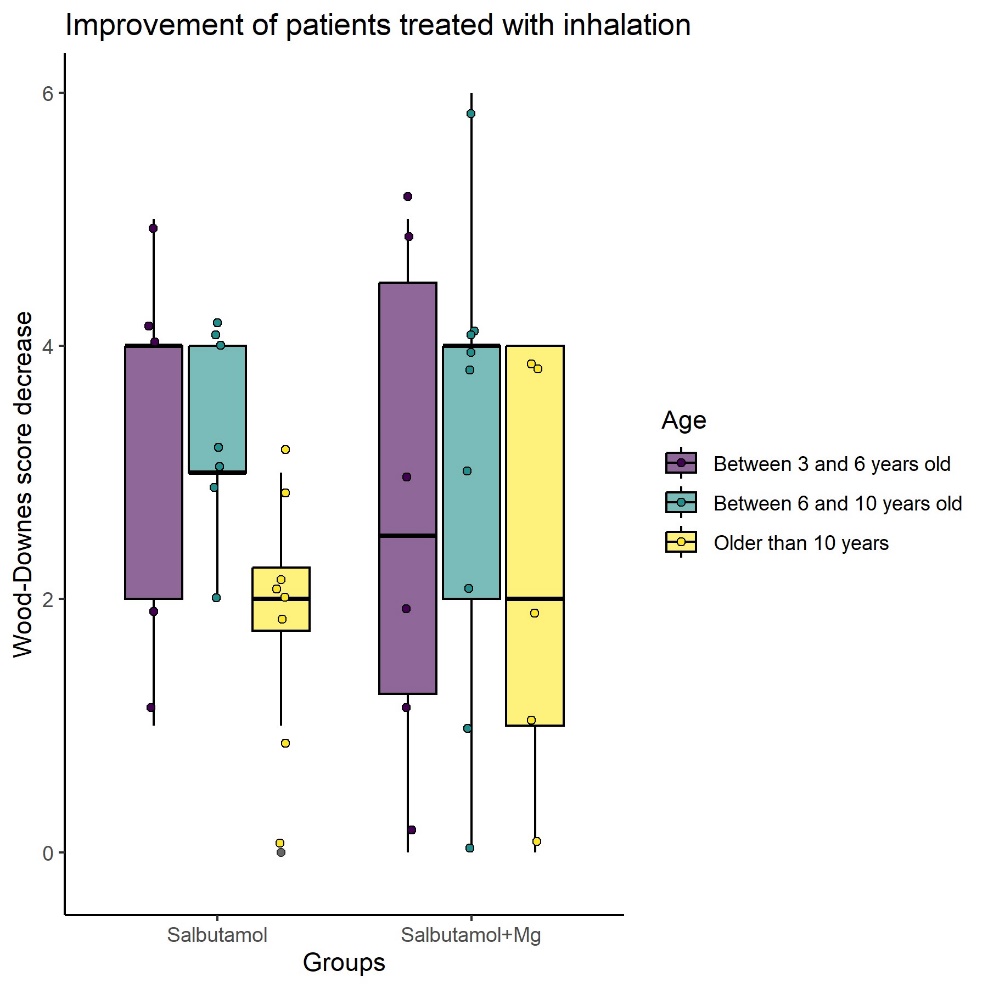


**Figure S5** Correlation between score and age > 10 years.


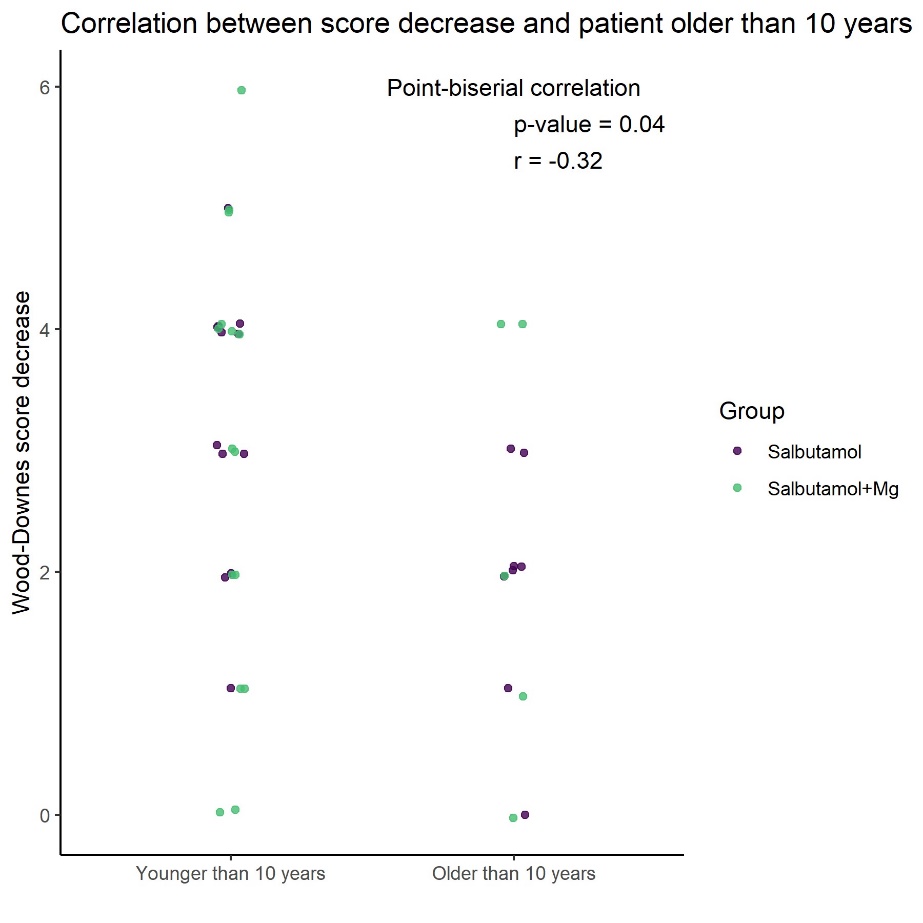


**Figure S6** Correlation between score and domiciliary use of Beclomethasone.


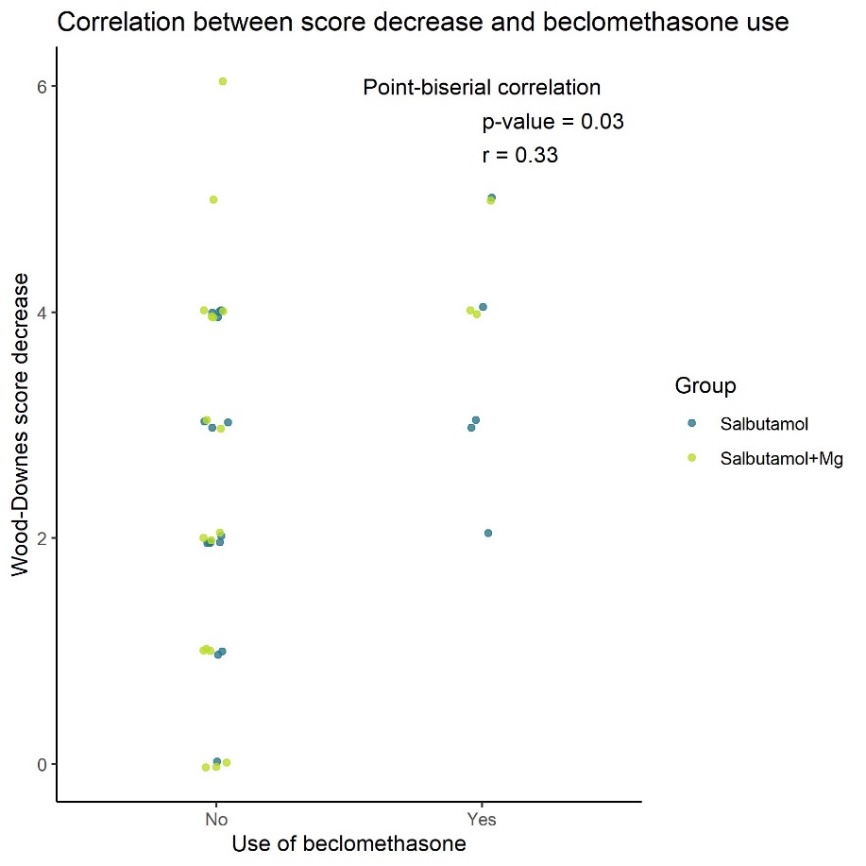


**Figure S7** Correlation between score and ICU admission.


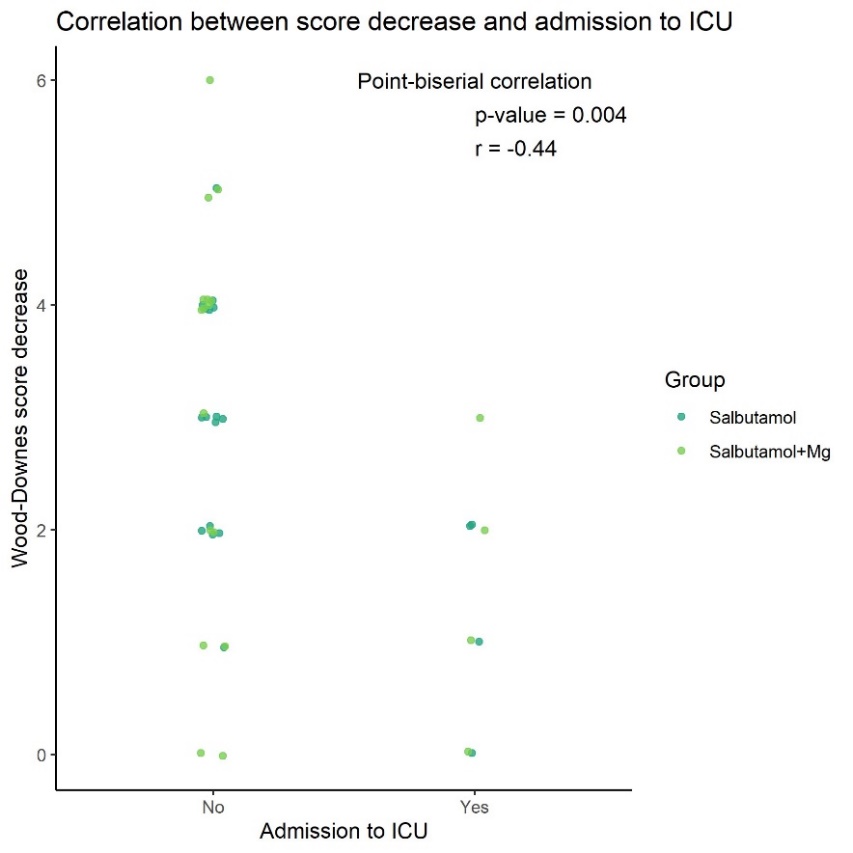

Supplement: Supplementary file 1 [file mmc1.docx]
